# Ocular surface health of the Finnish elderly population

**DOI:** 10.1111/aos.15130

**Published:** 2022-03-24

**Authors:** Ulla Aapola, Janika Nättinen, Ilona Suurkuukka, Jaakko Tuomilehto, Sirkka Keinänen‐Kiukaanniemi, Jouko Saramies, Hannu Uusitalo

**Affiliations:** ^1^ SILK Department of Ophthalmology Faculty of Medicine and Health Technology Tampere University Tampere Finland; ^2^ South Karelia Social and Health Care District South Karelia Finland; ^3^ Public Health Promotion Unit Finnish Institute for Health and Welfare Helsinki Finland; ^4^ Saudi Diabetes Research Group King Abdulaziz University Jeddah Saudi Arabia; ^5^ Department of International Health National School of Public Health Instituto de Salud Carlos III Madrid Spain; ^6^ Center for Life Course Health Research University of Oulu Oulu Finland; ^7^ Tays Eye Centre Tampere University Hospital Tampere Finland

**Keywords:** dry eye disease, epidemiology, ocular surface disease, prevalence

## Abstract

**Purpose:**

The aim of this study was to describe the clinical ocular surface characteristics in a population‐based sample of Finnish elderly people.

**Methods:**

This cross‐sectional study included 601 subjects (335 females, 266 males) born between the years 1933–1956 and living in Savitaipale, Finland. Ocular surface health was evaluated using a comprehensive set of diagnostic tests. Previous dry eye (DE) diagnosis and history of drug treatment of DE were also recorded. Differences between sexes were estimated with Wilcoxon rank sum test and Fisher's exact test.

**Results:**

Overall, 10% and 33% of people displayed signs of DE and ocular surface disease (OSD), respectively, and 30% had been previously diagnosed with DE and 36% used some form of drugs for DE. Men displayed more severe signs of meibomian gland dysfunction, blepharitis and conjunctival redness (p < 0.001), while women had higher scores in corneal staining (p = 0.005) and OSD Index (p < 0.001).

**Conclusion:**

Signs of OSD and DE are common among the Finnish elderly population. However, the diagnosis is affected by the diagnostic criteria used and significant differences exist between sexes. Although women were more frequently diagnosed with DE and OSD and experienced more ocular surface irritation, men had more often lid and meibomian gland‐related issues. The current diagnostic criteria of DE pose a risk of misclassifying men, who commonly display less severe symptoms in comparison with women yet exhibit more severe clinical signs associated especially with the lid margin.

## Introduction

A healthy ocular surface is vital for the health of the whole eye, good vision and quality of life. Any dysfunction in the ocular surface can lead to ocular surface diseases (OSDs), which comprise a collection of multifactorial diseases and most often cause tear film instability, and ocular surface tissue inflammation and damage. Estimates of OSD prevalence vary greatly as different diagnostic criteria and study sample characteristics (age, sex, location, etc.) are applied in studies (Stapleton et al. 2017). For example, dry eye disease (DE), which is the most frequently studied OSD, is estimated to affect 5–50% of the population depending on the study (Stapleton et al. 2017). Nordic epidemiology studies on OSD are scarce and over two decades old (Jacobsson et al. 1989; Bjerrum 1997). Therefore, the need for epidemiological studies is high, especially for studies where not only the subjective and objective OSD diagnostic variables are carefully assessed, but also participants' lifestyle, general health and socioeconomic status are examined and recorded.

In addition to difficulties in diagnostic criteria selection, differences between sexes and age groups are an important aspect in prevalence estimations. Age is a well‐known risk factor of OSD and DE, and previous articles have also pointed out differences in signs and symptoms between women and men (Paulsen et al. 2014; Sullivan et al. 2017; Borrelli et al. 2021; Nøland et al. 2021). Women have been found to be more affected by DE symptoms according to OSD Index (OSDI) questionnaire results, and recent studies have also observed that men are more prone to meibomian gland dysfunction (MGD) and conjunctival redness (Vehof et al. 2018; Borrelli et al. 2021). Whether these sex differences are applicable in all age groups and locations is still unclear.

The population‐level information on the variation within different ocular surface variables, among women and men, can help identify, which clinical aspects are more frequent in different patient groups. This knowledge enables efficient early intervention, before the development of OSD. Therefore, our aim was to examine the ocular surface state and health in the cohort of a Finnish population aged 60 years or over by carefully evaluating a wide selection of clinical ocular surface signs and symptoms among all participants and between women and men. We also compared these findings to the prevalence of OSD and DE in this cohort using different diagnostic criteria. To our knowledge, this is the first population‐based study conducted on Finnish population.

## Methods

### Study structure and participants

This cohort study is part of a larger longitudinal health and lifestyle study (data collected in years 1996–1999, 2007–2008 and 2018–2019). All habitants of Savitaipale, a rural municipality in south‐eastern Finland, who were born between the years 1933–1956 (1508 people in total), received an invitation letter to the initial Savitaipale Study on 27 May 1996. The participants were then followed up with re‐examinations 10 and 22 years after the baseline study. In the final 22‐year follow‐up visit in 2018–2019, the target population consisted of 906 participants, and of these, 704 participated in the follow‐up clinical examinations and 601 participated also in the ophthalmic examination, which is the focus of this study. Further details of the study design have been recently published (Saramies et al. 2021). Written informed consent was obtained from participants before the study. This study was conducted in compliance with the principles of the Declaration of Helsinki and was approved by the Ethics Committee of the Helsinki University Hospital (HUS/2203/2018).

### Measurements

An ophthalmologist (IS) investigated the participants thoroughly during their visit. Basic health data including previous DE diagnosis and drugs used were collected, and a thorough ophthalmologic evaluation was performed, including analysis of refraction, visual acuity, slit‐lamp examination, measurement of the intraocular pressure and indirect ophthalmoscopic evaluation of the retina. Various ocular surface health parameters were assessed. These included Schirmer's *I* test, fluorescein tear break‐up time (FTBUT), non‐invasive tear break‐up time (NIBUT), corneal fluorescein (FLUOstrips, Contacare Ophthalmics and Diagnostics, Gujarat, India) and conjunctival Lissamine green (I‐Dew Green, Entod Research Cell UK Ltd, London, UK) staining, conjunctival redness, blepharitis, MGD and OSDI. Conjunctival redness was graded by using conjunctival redness reference photographs (scale 0–4), blepharitis and MGD by using the Efron scale (0–4) and corneal and conjunctival staining by using the Oxford grading (0–5). Ocular surface health parameters of both eyes were examined, and the worse eye was selected for the statistical analysis based on Schirmer's *I* test, FTBUT and corneal staining.

### Diagnostic criteria

The presence of OSD and DE was investigated by evaluating symptoms and clinical signs. For DE, diagnosis slightly modified guidelines of the DE Workshop (DEWS) 2017 report (Wolffsohn et al. 2017) were used: the OSDI score of ≥13 accompanied by at least one of the following signs: NIBUT <10, corneal (fluorescein) staining grade >0 or conjunctival (Lissamine green) staining grade >0. OSD was diagnosed if at least two of the following signs or symptoms were present: NIBUT <10, Schirmer *I* test <10, OSDI ≥13 or corneal (fluorescein) or conjunctival (Lissamine green) staining grade >0.

### Statistical analysis

All statistical analyses were performed with R (version 4.1.2). Differences in signs and symptoms between sexes were evaluated using Wilcoxon rank sum test for ordinal data and non‐normally distributed data (normality tested with Shapiro–Wilk test). Fisher's exact test was used to evaluate differences in DE and OSD counts between sexes. The effects of clinical signs on DE medication use frequency were estimated using Kruskal–Wallis rank sum test for continuous variables and Fisher's exact test for discrete variables. The threshold of statistical significance in all analyses was p‐value <0.05.

## Results

The ophthalmic examinations were conducted between January and June 2019, and the study sample consisted of 601 participants (335 women and 266 men). The mean age was 72.1 ± 6.3 years (ranging from 62 to 86 years), and there was no statistically significant difference in age between sexes.

According to the clinical ocular surface signs and their associated thresholds (Table 1), 16% of participants displayed mild, moderate or severe signs of MGD and 17% signs of blepharitis (Efron scale ≥ 2). In addition, a large proportion of participants had traces of MGD or blepharitis (Efron scale = 1), 38% and 44% respectively (data not shown). Corneal and conjunctival staining (Oxford scale ≥ 1) were observed in 29% and 12% of participants respectively. Over half (55%) of participants had signs of conjunctival redness, 31% had Schirmer's test value below 10 mm, and 27% had NIBUT value below 10 seconds. According to OSDI scores, 12% of participants had mild, 6% moderate, and 3% severe DE symptoms.

**Table 1:** Prevalence of separate ocular surface signs and symptoms among the study subjects.

Clinical sign/symptom	Unit of measure (range)	Threshold	Signs/symptoms according to threshold, count (%)	
			No	Yes
MGD	Efron scale (0–4)	≥2	507 (85)	93 (16)
Blepharitis	Efron scale (0–4)	≥2	500 (83)	100 (17)
Conjunctival redness	Reference photographs' scale (0–4)	≥2	273 (46)	327 (55)
Corneal staining	Oxford scale (0–5)	≥1	424 (71)	176 (29)
Conjunctival staining	Oxford scale (0–5)	≥1	528 (88)	71 (12)
OSDI	OSDI score (0–100)	≥13	473 (79)	127 (21)
	Mild	13–22		74 (12)
	Moderate	23–32		36 (6)
	Severe	≥33		17 (3)
NIBUT	s (0–10)	<10	436 (73)	162 (27)
Schirmer's *I* test	mm (0–35)	<10	411 (69)	188 (31)

MGD = meibomian gland dysfunction, NIBUT = non‐invasive tear break‐up time, OSDI = Ocular Surface Disease Index.

**Fig. 1:**
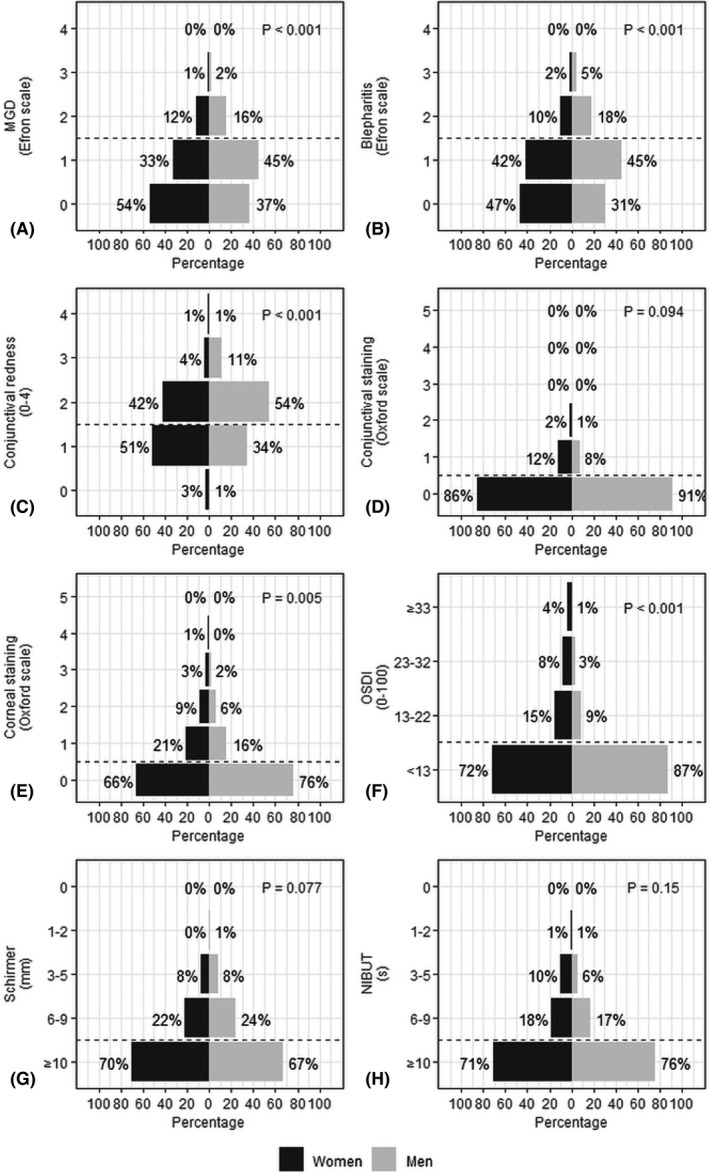
Prevalence of clinical ocular surface findings by sex. The horizontal dashed lines indicate the diagnostic thresholds used in this study for each clinical finding. Comparisons between women and men were made using the Wilcoxon rank sum test. MGD = meibomian gland dysfunction, NIBUT = non‐invasive tear break‐up time, OSDI = Ocular Surface Disease Index.

**Fig. 2:**
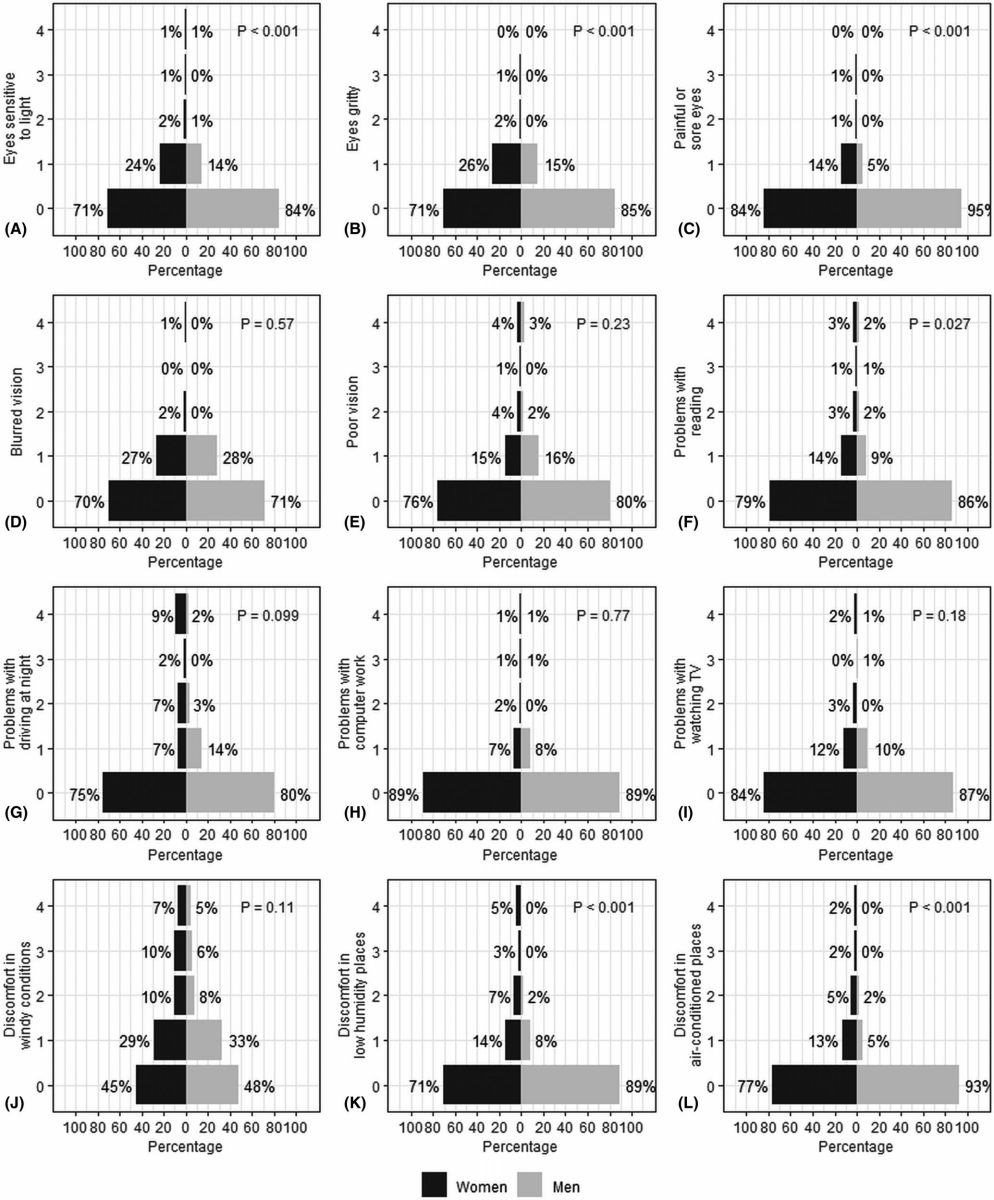
Prevalence of ocular surface symptoms by sex. The questions are based on the Ocular Surface Disease Index (OSDI) questionnaire questions. Comparisons between women and men were made using the Wilcoxon rank sum test. For visual sensations (A–E), problems with eyes (F–I), discomfort in specific situations (J–L), the scoring was following: 0 = none of the time, 1 = some of the time, 2 = half of the time, 3 = most of the time, 4 = all the time.

Overall, the most frequent ocular surface issue in both sexes was conjunctival redness. Men had more frequently signs of MGD, blepharitis and conjunctival redness (p < 0.001, Wilcoxon rank sum test), in comparison with women, while women displayed higher corneal staining scores (p = 0.005) (Fig. 1). Women also tended to have higher OSDI scores than men (p < 0.001) and women found that their eyes were more frequently sensitive to light, gritty and painful (Fig. 2). Women also experienced more frequently problems while reading (p = 0.03) and discomfort in low humidity and air‐conditioned places (p < 0.001) in comparison with men. Among both sexes, over one‐fourth of participants experienced blurred vision at least some of the time, and over half experienced discomfort in windy conditions.

**Fig. 3:**
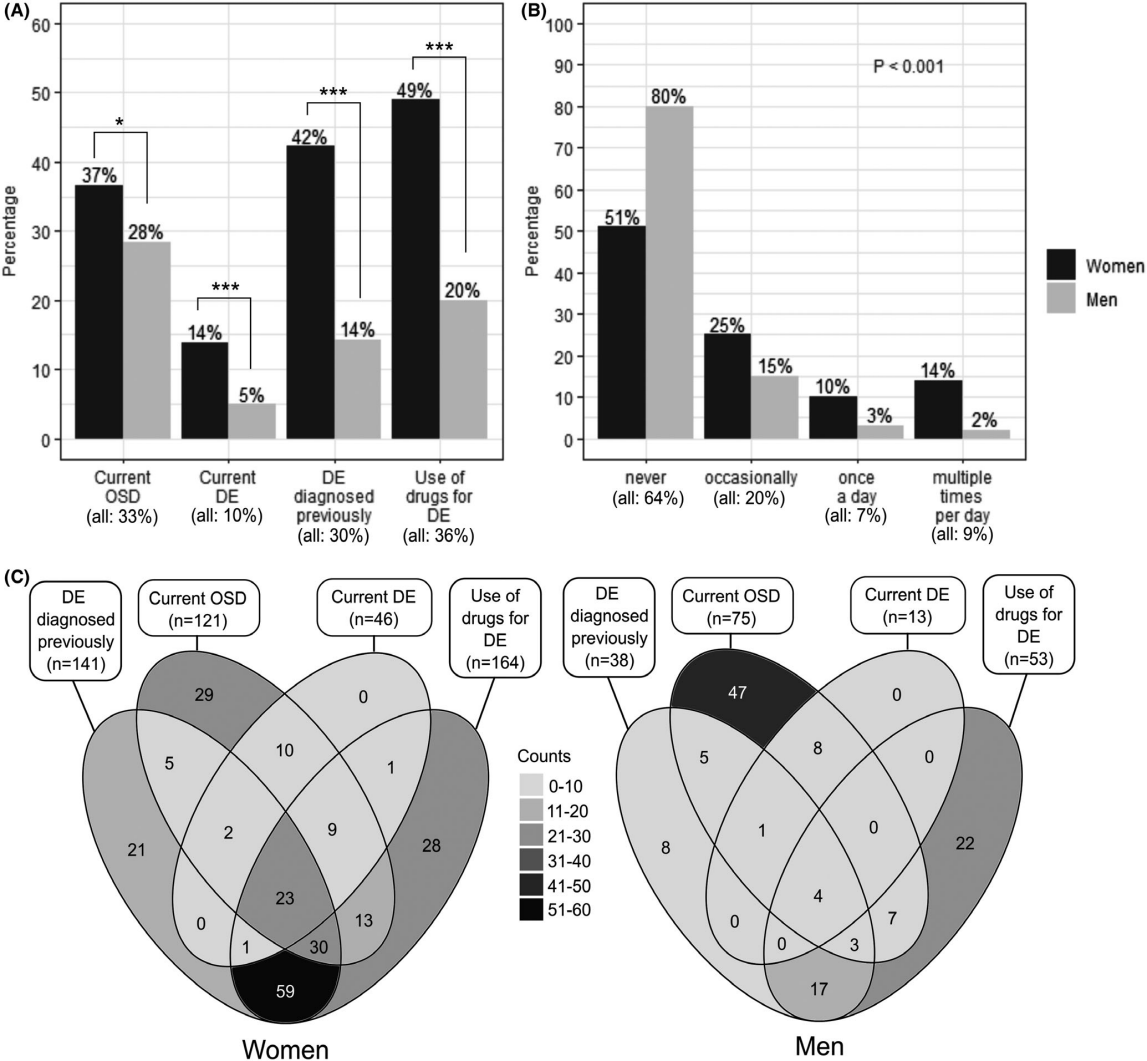
Prevalence of dry eye (DE) and ocular surface disease (OSD) and use of drugs for DE. (A) Frequency of current OSD and DE, as well as previously diagnosed DE and use of drugs for DE by sex. (B) Frequency of drugs used for DE by sex. (C) Venn diagrams of the counts overlapping between current OSD, DE, previously diagnosed DE and use of drugs for DE. Statistical comparisons between women and men (A, B) were made using Fisher's exact test. *p < 0.05, ***p < 0.001.

According to our diagnostic criteria for DE and OSD, 59 participants (10%) had signs or symptoms qualifying for DE diagnosis and 196 (33%) for OSD diagnosis (Fig. 3). Of the participants, 179 (30%) had been previously diagnosed with DE and 217 (36%) had used drugs for DE.

When comparing between sexes, the frequency of previously diagnosed DE and use of drugs for DE, as well as current DE and OSD according to our diagnostic criteria were all significantly higher among women (Fig. 3A). Almost half of all women had used drugs for DE, while men had either never used or used only occasionally these drugs (Fig. 3B). Based on Venn diagrams (Fig. 3C), many women had been previously diagnosed with DE and used drugs for DE. Other large groups consisted of women, who currently displayed OSD signs, yet had no treatment or previous diagnosis and those who currently used DE medication and still displayed signs of OSD and, in some cases, DE. Many men had signs of some form of OSD yet did not have DE diagnosis or signs nor did they use drugs for DE.

**Fig. 4:**
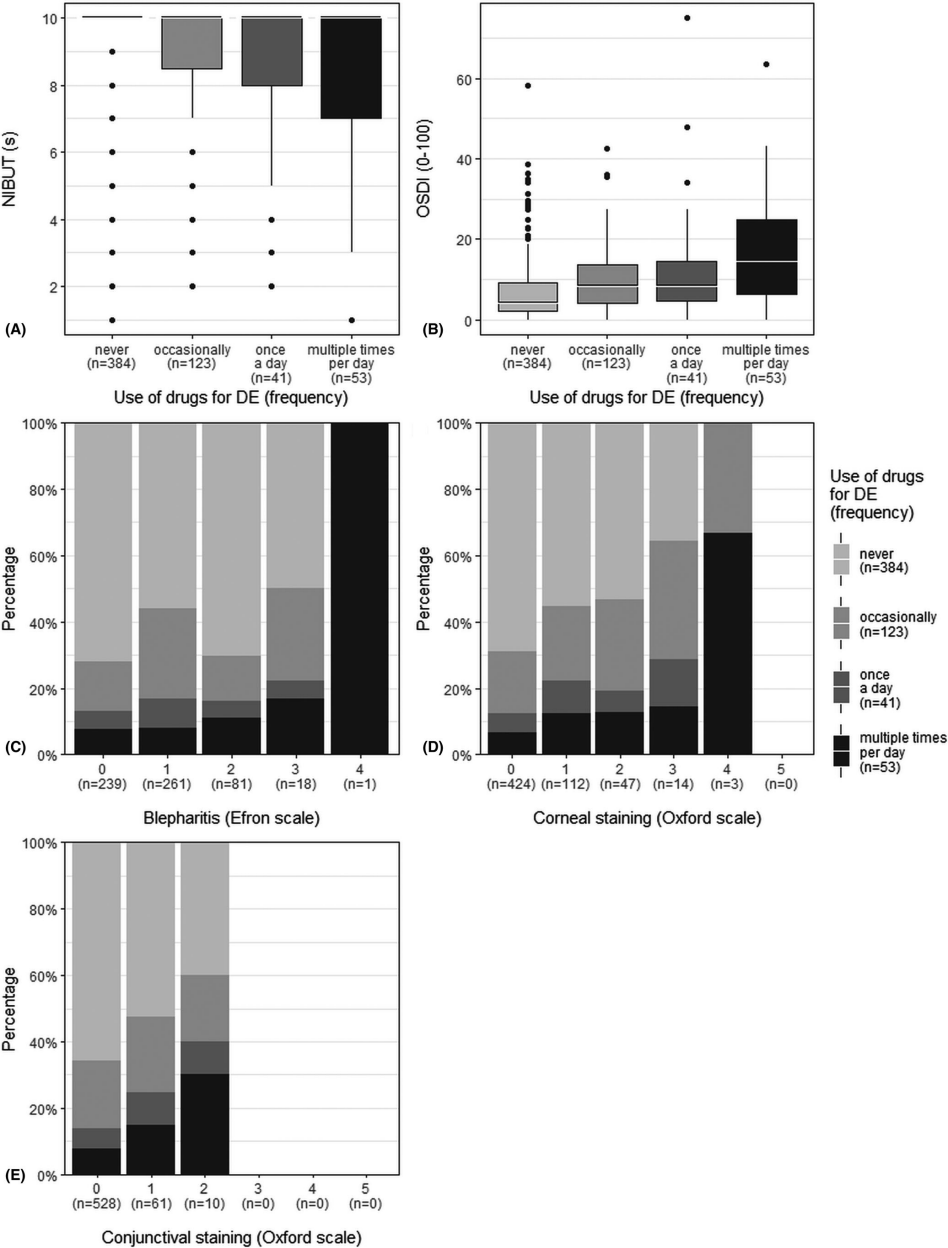
Frequency of clinical signs and symptoms associated with the use of drugs for dry eye (DE). (A, B) Non‐invasive tear break‐up time (NIBUT) and Ocular Surface Disease Index (OSDI) scores were associated with the use of drugs for DE according to results obtained using Kruskal–Wallis rank sum test (p = 0.01 and p < 0.001, respectively). (C–E) Increased blepharitis, corneal staining and conjunctival staining scores were also associated with more frequent use of drugs for DE according to results obtained using Fisher's exact test (p = 0.003, p = 0.002 and p = 0.4, respectively).

The frequency of DE drug use (never, occasionally, once a day and multiple times a day) was associated with the levels of several clinical signs (Fig. 4). Decreased NIBUT and increased OSDI, blepharitis, corneal staining and conjunctival staining were associated with more frequent use of DE drugs. In addition, there were differences between sexes; decreased NIBUT and increased conjunctival redness were associated with an increased frequency of the use of drugs for DE among women only (p = 0.026, Kruskal–Wallis rank sum test, and p = 0.002, Fisher's exact test, respectively), while increased scoring in MGD and corneal staining were associated with an increased frequency of the use of drugs for DE among men (p = 0.006 and p = 0.03, Fisher's exact test, respectively) (for visualizations, see Fig. S1). In both sexes, the use of drugs for DE was more frequent when participants had increased OSDI and blepharitis scores.

## Discussion

As Tear Film & Ocular Surface Society's DE Workshop II (TFOS DEWS II) Epidemiology Report was published in 2017 (Stapleton et al. 2017), there has been a growing interest in collecting data on the prevalence and risk factors of DE in different countries. However, in most of the countries, including Finland, updated epidemiologic data on DE are still lacking (Erickson et al. 2020). In addition, the available estimates of DE vary markedly ranging from 5% to 50% due to varying diagnostic criteria and sample characteristics, such as age, sex and ethnicity (Stapleton et al. 2017). This complicates comparisons of the prevalence estimates among studies. In the present study, one ophthalmologist measured both subjective and objective clinical variables in all participants in order to evaluate comprehensively and consistently the ocular surface health in a population‐based sample of the elderly Finnish population. The subjective DE/OSD were evaluated using OSDI questionnaire scores, self‐reported use of drugs for DE and previous DE diagnosis, while thorough clinical evaluations of the ocular surface signs were performed to assess the ocular surface status objectively.

The prevalence of DE/OSD varied notably depending on the criteria used. According to TFOS DEWS II‐based symptoms and signs criteria, the prevalence of DE in this elderly Finnish study population was 10%, but when more relaxed criteria were applied (*i.e*. signs with or without symptoms), the prevalence increased to 33%. [Correction added on 05 May 2022, after first online publication: The increased prevalence percentage was corrected in the preceding sentence.] Compared with other recent prevalence studies applying TFOS DEWS II‐based symptoms and signs criteria for elderly populations, the 10% DE prevalence in elderly Finnish population seem to be relatively low. In New Zealand, the prevalence of DE reported among elderly people aged 60 years or over was 47% (Wang et al. 2020), in Indonesia, among people aged 50–80 years 22.5% (Noor et al. 2020) and in Palestine, in people aged 45 years or over as high as 70.7% (Shanti et al. 2020). All participants in our study lived in a rural countryside community where environmental hazards are rare; this may partly explain the relatively low DE prevalence in this population.

In our study, OSDI scores (i.e. symptoms‐only criteria) indicated that 21% of the participants had experienced DE symptoms, 36% of them reported the previous use of drugs for DE, and 30% had been previously diagnosed with DE. Many of the previous population studies have relied solely on the questionnaire data and thus resemble our symptoms‐only criteria. For example, in relation to similar age groups in two North American symptom‐based studies, Beaver Dam Eye Study (Moss et al. 2000) and a population‐based survey in Ontario, Canada (Caffrey et al. 2019), the prevalence was 15–20% and 23%, respectively, indicating similar findings to ours. However, some studies from the United States implementing similar criteria have reported a prevalence below 10% (Schaumberg et al. 2003; Schaumberg et al. 2009). In Europe, the Lifelines Cohort Study in the Netherlands estimated the DE prevalence in the elderly to be below 15% (Vehof et al. 2021), while in France, the estimate was well over 30% (Ferrero et al. 2018). In China, the prevalence of DE by symptoms has been reported to be 31% in people aged 5–89 years (Song et al. 2018). The large range in prevalence in symptom‐based studies can be expected as they focus solely on questionnaire or register data, which are never free of biases; questionnaire data can exclude people with asymptomatic DE and registers usually have data only from people already managed by health care professionals for the disease.

Consistent with the previous studies, our data revealed that OSDs and symptomatic DE were more common in women than men (Sullivan et al. 2017). Especially, elderly women have been found an increased risk of developing OSD and DE; this is thought to be due to their altered hormone signalling affecting the lacrimal and meibomian gland secretions (Sullivan et al. 2006). In relation to symptoms experienced by the participants, detailed comparison of sex differences in OSDI revealed that women were more sensitive to light and experienced more ocular pain than men. It has been suggested that pain works partly through different pathways between women and men (Sorge et al. 2015; Gregus et al. 2021). Supporting findings arise from the recent study by Li and Lin (2019), where increased DE symptoms of women were associated with a greater pain sensitivity, whereas in men, there was no such association. In the future, studies on sex differences in self‐reported symptoms and potential differences in corneal sensitivity might help to understand better these findings. On the contrary, according to our findings, men had worsened ocular surface signs in comparison with women. Especially, signs related to eye lid health showed a significantly worse status for men. In Japan, Arita et al. 2019 carefully studied both MGD and DE signs and symptoms in a Japanese population and concluded that prevalence of DE (33.4%) was associated with a female sex, and the prevalence of MGD (32.9%) with a male sex, while coexistence of both DE and MGD was reported to be 12.9% among all participants (Arita et al. 2019). Symptoms and signs of DE and MGD are overlapping; with eye examinations used in our study, it was difficult to distinguish whether men had MGD or DE, or both. This is an issue that should be addressed in future studies.

Consistent with our findings and previous studies, OSD and DE symptoms were affecting the everyday life in women more frequently than in men, and women also reported a more frequent use of drugs for OSD and DE than men (Schaumberg et al. 2013; Malet et al. 2014; Ferrero et al. 2018; Caffrey et al. 2019). In our study, approximately half of women reported history of the use of drugs for DE while in men the corresponding frequency was one‐fifth. Also, it appeared that a large number of men displayed current signs of OSD, but did not use any drugs for it, indicating that, compared with women, OSD in men was either more frequently asymptomatic or they had not searched treatment for the condition despite the symptoms. The difference in frequency of the use of drugs for DE may be explained also by the fact that many of the findings, which were higher in women than men such as OSDI and corneal staining, were associated with the use of drugs for DE.

The current study has some limitations. The tear film osmolarity was not measured, which meant that we were unable to use the specific TFOS DEWS II DE criteria in our analyses. This could affect the DE prevalence observed and should be considered when comparing these results to other population‐based prevalence estimates. In addition, in this study, we did not divide the people with OSD and DE to further subcategories, such as aqueous‐deficient and evaporative DE. Further categorization of the DE and OSD patients would be interesting, and our aim is to study this further in the future.

## Conclusions

In conclusion, the prevalence of OSD and DE among elderly Finnish population is estimated to be approximately 10% to 33% depending on the criteria used. [Correction added on 05 May 2022, after first online publication: The prevalence % range was corrected in the preceding sentence.] Differences between sexes are evident; women display more frequently OSD and DE symptoms, which can affect their wellbeing and results in an increased use of drugs for DE, while men display worse clinical, especially lid‐function related signs. These differences can be addressed in clinical work and in further research in order to understand better, what are the clinical and biological functions driving these differences.

## Supporting information


**Fig. S1.** Clinical signs and symptoms associated with dry eye (DE) medication use frequency by sex. (A, B) Boxplots displaying the DE medication use frequency by sex and its association with non‐invasive tear break‐up time (NIBUT) and Ocular Surface Disease Index (OSDI). (C–F) Stacked bar plots showing the frequency of DE drug use by sex and its association with blepharitis, Meibomian gland dysfunction (MGD), conjunctival redness and corneal staining. The p‐values were obtained using Kruskal–Wallis rank sum test (A, B) and Fisher's exact test (C–F).
