# Supplementary figures and images for: Ocular surface health of the Finnish elderly population
Source: Acta Ophthalmol. 2022 Mar 24;100(8):894–902. doi: 10.1111/aos.15130 (PMC9790390; doi:10.1111/aos.15130)

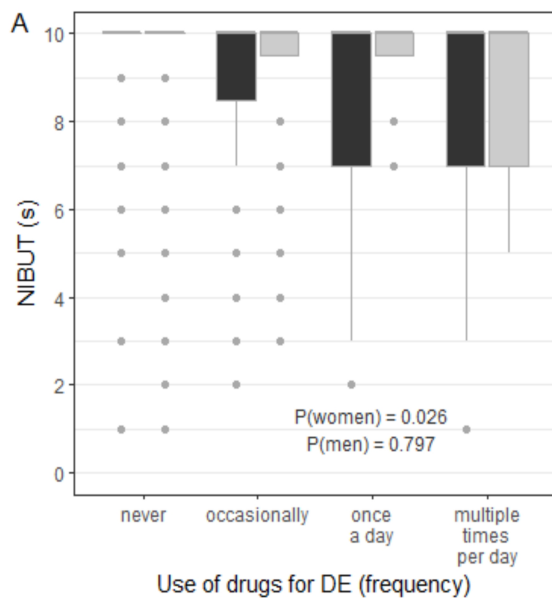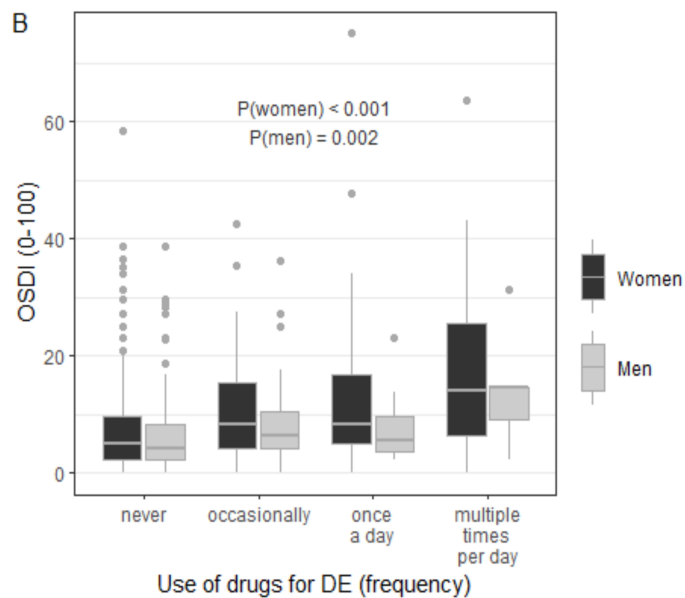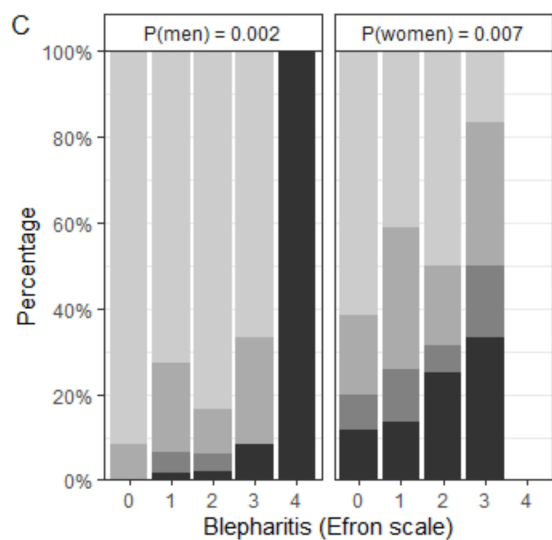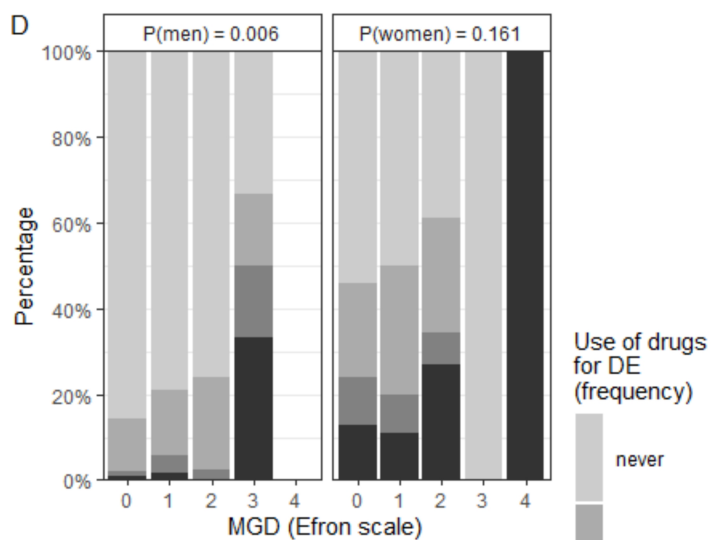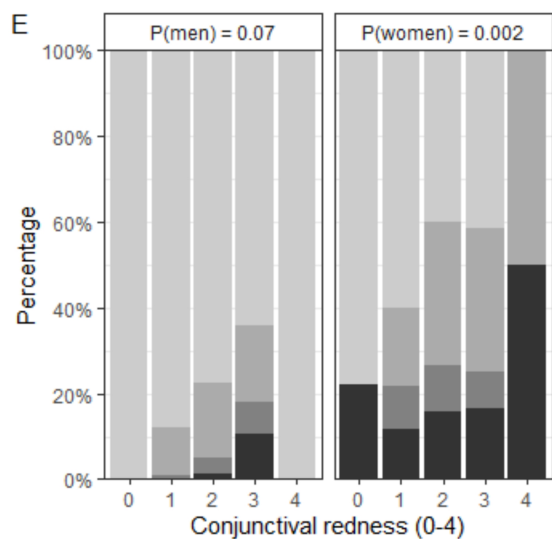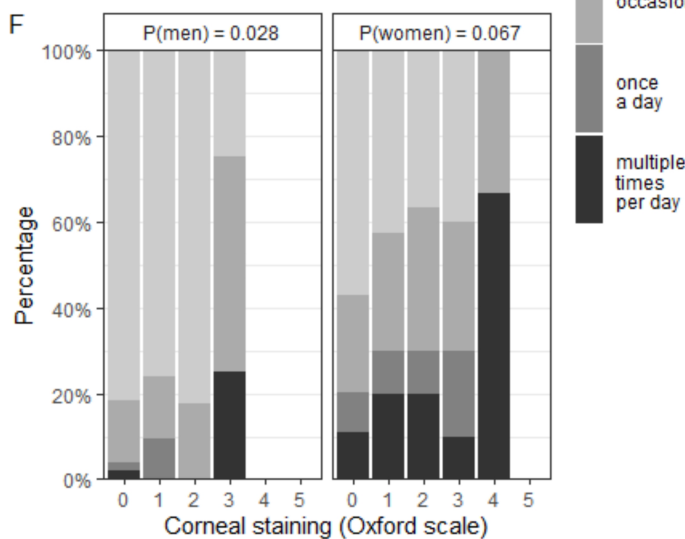

Supplement: Supplementary file 1 — Fig. S1. Clinical signs and symptoms associated with dry eye (DE) medication use frequency by sex. (A, B) Boxplots displaying the DE medication use frequency by sex and its association with non‐invasive tear break‐up time (NIBUT) and Ocular Surface Disease Index (OSDI). (C–F) Stacked bar plots showing the frequency of DE drug use by sex and its association with blepharitis, Meibomian gland dysfunction (MGD), conjunctival redness and corneal staining. The p‐values were obtained using Kruskal–Wallis rank sum test (A, B) and Fisher's exact test (C–F). [file AOS-100-894-s001.pdf]
